# 3D ED: Accessible, robust and accurate

**DOI:** 10.1107/S2052252526001855

**Published:** 2026-02-24

**Authors:** Stéphanie Kodjikian

**Affiliations:** a Université Grenoble Alpes, CNRS, Grenoble INP, Institut Néel, 38000Grenoble, France

**Keywords:** three-dimensional electron diffraction, 3D ED

## Abstract

Now a common technique in crystallography, three-dimensional electron diffraction (3D ED) is on track to bridge the gap with X-ray diffraction in terms of structural accuracy.

Understanding the organization of atoms in matter is essential to comprehension of the origin of a material’s chemical, physical or biological properties. However, for a long time, the accuracy of structures obtained by electron diffraction was compromised by the very strong interaction of electrons with matter and its consequences on the intensity of diffracted beams (in the case of multiple diffraction, the diffracted intensity is no longer proportional to the square modulus of the structure factor). Another major drawback of electron diffraction was the imprecise determination of lattice parameters.

Recent decades have seen spectacular technical developments in transmission electron microscopy: beam precession (Vincent & Midgley, 1994 ▸), tomography experiments (Kolb *et al.*, 2007 ▸; Kolb *et al.*, 2008 ▸), fast and sensitive pixelated detectors, and cryogenic experiments. Thanks to these major advances, electron diffraction data can finally be measured reliably, enabling the calculation of a structural model, which can then be refined to take dynamic effects into account through the development and constant updating of dedicated crystallography software (Palatinus, 2011 ▸; Petřiček *et al.*, 2014 ▸; Palatinus *et al.*, 2019 ▸). These methods are commonly grouped under the generic term ‘Three-dimensional electron diffraction’ (3D ED) (Gemmi *et al.*, 2019 ▸). They are mainly performed on transmission electron microscopes (TEMs), but more recently also on dedicated equipment: electron diffractometers (Rigaku’s Synergy-ED and ELDICO’s ED-1). 3D ED is thus becoming a common technique in crystallography.

However, each group uses its own methods, often developed according to the equipment available, so the term ‘3D ED’ actually covers many variants. This raises two questions: how accurate can structures obtained by 3D ED be expected to be? Is there a preferred configuration and/or methodology?

To answer these two questions, the first 3D ED round-robin challenge was organized as part of the NanED doctoral network (Gemmi *et al.*, 2026 ▸). The principle of a round-robin challenge is simple: ask several groups to analyze the same sample without any preliminary knowledge. In this case, seven groups already experienced in 3D ED solved the structure of three compounds [epidote (see Fig. 1 ▸), natrolite, ibuprofen], and the structures obtained were validated by comparison with reference structures. The samples were selected to assess the robustness of 3D ED on the following main challenges: calculating the occupancy rate of mixed sites, locating hydrogen atoms and obtaining the absolute structure, including for a compound sensitive to irradiation (ibuprofen).

At the end of the round robin, the main conclusions of Gemmi *et al.* (2026 ▸) are as follows:

(1) *Equipment/methodology*. All groups succeeded in determining the structure of the two inorganic compounds, confirming that 3D ED can be applied regardless of the technical configuration used. However, obtaining the structure of ibuprofen required cryogenic and low-dose conditions.

(2) *Accuracy of crystallographic structures*. For the two inorganic compounds, the average accuracy of atomic positions (0.03 Å after kinematical refinement) was improved to 0.01 Å by dynamical refinement. The best precision was remarkably low: 0.004 Å. For beam-sensitive ibuprofen, the most accurate structure was obtained after kinematical refinement, by merging data sets from different crystals (average accuracy in atomic positions = 0.05 Å).

According to the authors, merging several data sets reduces the dynamical scattering effects in the data. This strategy proves successful when the crystal quality is too poor for dynamical refinement to improve the accuracy of the structure. Nevertheless, dynamical refinement is still relevant if the number of datasets is low.

(3) *Relevance of R values for 3D ED*. The correlation between the accuracy of atomic positions and low *R* values is verified in the inorganic compounds (but not as clearly established in the case of ibuprofen).

(4) *Absolute structure*. Dynamical refinements allow two enantiomorphs to be easily distinguished both in organic and inorganic compounds.

(5) *Precision and accuracy of lattice parameters*. These can be improved by accurately calibrating the detector and effectively correcting distortions inherent in the use of electron microscopes.

In addition to these conclusions, readers will find in the paper of Gemmi *et al.* (2026 ▸) and its supplementary information an educational description of the main instrumental and methodological aspects used in 3D ED: sample preparation; equipment, experimental conditions and acquisition methods; and data-processing procedures with descriptions of the most commonly used software [much of which has been developed for X-ray diffraction (XRD)].

In summary, particularly well suited to the study of nanomaterials and easy to access, electron diffraction has become indispensable in nanoscale crystallography: powder compounds (large lattice, low symmetry), polyphase samples *etc*. With a level of precision on atomic positions now close to that of XRD, and even superior performance for light atoms (Palatinus *et al.*, 2017 ▸; Gemmi *et al.*, 2026 ▸), 3D ED promotes synergies between communities: X-ray crystallographers are joining forces with TEM and electron diffractometer platforms to conduct their first 3D ED experiments, while material science microscopists are collaborating with their colleagues in the life sciences to benefit from their expertise in vacuum- and radiation-sensitive samples (Yörük *et al.*, 2025 ▸). In this context, there is no doubt that the work reported in the current issue of *IUCrJ* by Gemmi *et al.* (2026 ▸) will serve as a reference for a wide audience of experimenters wishing to learn about 3D-ED or perfect their practice.

## Figures and Tables

**Figure 1:**
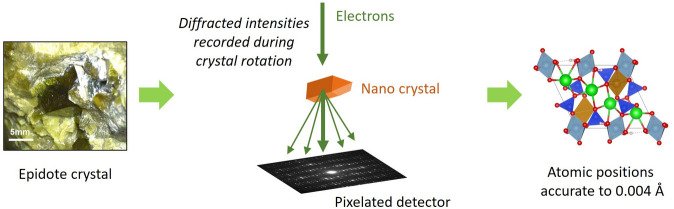
From crystal to structure solution. The application of 3D-ED to epidote has enabled remarkable accuracy to be achieved for certain atomic positions (0.004 Å). Images of the epidote crystal and structure model reproduced from Gemmi *et al.* (2026 ▸).
